# Multiphysics Modeling and Analysis of a Solar Desalination Process Based on Vacuum Membrane Distillation

**DOI:** 10.3390/membranes11060386

**Published:** 2021-05-25

**Authors:** Benjamin N. Shuldes, Mona Bavarian, Siamak Nejati

**Affiliations:** Department of Chemical and Biomolecular Engineering, Othmer Hall, University of Nebraska-Lincoln, Lincoln, NE 68508, USA; bshuldes2@unl.edu (B.N.S.); mona.bavarian@unl.edu (M.B.)

**Keywords:** multiphysics, vacuum membrane distillation, exergy analysis, solar thermal desalination

## Abstract

A hollow fiber vacuum membrane distillation (VMD) module was modeled using finite element analysis, and the results were used to conduct an exergy efficiency analysis for a solar-thermal desalination scheme. The performance of the VMD module was simulated under various operating conditions and membrane parameters. Membrane porosity, tortuosity, pore diameter, thickness, and fiber length were varied, along with feed temperature and feed configuration. In all cases, polarization phenomena were seen to inhibit the performance of the module. Under VMD operation, polarization of salt concentration was seen to be the main determining factor in the reduction of permeate flux. Within the boundary layer, salt concentration was seen to rapidly increase from the feed mass fraction of 0.035 to the saturation point. The increase in salt concentration led to a decrease in saturation pressure, the driving force for separation. Charging the feed into the shell instead of the lumen side of the membranes resulted in a further decrease in permeate flux. It is shown that adding a baffling scheme to the surface of the fibers can effectively reduce polarization phenomena and improve permeate flux. Increasing the overall recovery ratio was seen to increase the exergy efficiency of the system. Exergy efficiency was seen to have almost no dependency on membrane parameters due to the low recovery ratio in a single pass and the high heating duty required to reach the desired temperature for the feed stream.

## 1. Introduction

As more people come to live in water-strained areas, water resources struggle to accommodate personal, industrial, and agricultural needs [1]. The continued stress placed on natural freshwater resources damages ecosystems and draws off an already limited supply of freshwater. In countries like Singapore that have few natural freshwater sources, seawater desalination is required for achieving economic independence [2]. Many cities on the Persian Gulf, like Abu Dhabi, have long invested in desalination technology [3]. However, as populations continue to rise, traditional desalination practices place a larger burden on the energy infrastructures and become less desirable. This burden can be alleviated by utilizing membrane-based separation and powering desalination plants with renewable energy [4] or by introducing novel, energy-efficient, and sustainable separation schemes for increasing water recovery. Along these lines, reverse osmosis (RO) plants operating on photovoltaics [5] and solar-thermal integrated flash distillation have already been explored [6]. Nonetheless, operational maintenance remains a bottleneck, and the need to find other integration schemes to tap into renewable energy for desalination is still an ongoing work.

Membrane distillation (MD) is a developing technology that is now being widely investigated for its potential use in water desalination [7]. In practice, it can be used in conjunction with RO to concentrate brine and increase the overall water recovery. In MD, an air-filled, non-wetting membrane provides a barrier through which water vapor can be transferred when there is a driving force. Vacuum membrane distillation (VMD) is a membrane distillation scheme in which a partial vacuum is applied to one side of the membrane while maintaining a pressure drop that is below the liquid entry pressure for the membrane [8,9]. In MD, permeate flux is limited by mass transfer in the membrane. Because of its extremely low resistance to mass transfer, VMD, among all MD configurations, is expected to provide the highest permeate vapor flux. In theory, VMD can be utilized to achieve the highest water recovery rate of all MD schemes [9].

Given the potential of VMD for achieving a high recovery ratio, it can be seen as a candidate process for integration into desalination schemes [8]. Similar to other desalination schemes that process high salinity feed water at a high rate, in MD, scale formation at the membrane/feed interface is known to be a major challenge [7,10,11]. Scaling is exacerbated by polarization in which heat and mass transfer through the membrane results in an increase in salt concentration and a decrease in temperature along the membrane/feed interface. Recent advances in membrane fabrication have led to the realization of membrane materials that can operate at high salt concentrations [10,12]. We have previously demonstrated the fabrication of hollow fiber membranes (HFMs) that can handle high salinity solutions with a salt concentration near saturation [10]. Additionally, the application of physical methods, such as the addition of baffling or spacers for reducing polarization can be utilized to further mitigate scaling [13,14]. Yet another problem with VMD is the high thermal energy requirement associated with it [7,15]. A significant amount of thermal energy is needed to evaporate water. To circumvent this challenge, it is desirable to use “free” and “clean” forms of energy. Solar energy is the natural candidate for this purpose; it is abundantly available and can readily be converted into thermal energy for use in MD [15]. The remaining challenge is then the design of schemes that can achieve the desired recovery ratio and make effective use of renewable energy sources. To do so, it is important to understand the effects of various membrane parameters and transport phenomena within a module.

Here, we present a scheme for solar thermal membrane distillation utilizing an HFM module operated under a VMD configuration. Around this scheme, an exergy analysis was performed to determine the effect of membrane properties on the process. To gain particular insight into the role of membrane parameters, a finite element model was developed for an HFM module. We investigate the heat, mass, and momentum transfer phenomena present within the module. This model demonstrated the role of membrane parameters on permeate flux. Additionally, it revealed the role of temperature and concentration polarization as the limiting phenomena influencing the performance of the system. To mitigate the effects of these polarization phenomena a scheme was devised to break the boundary layer in which these polarization phenomena occur.

## 2. Materials and Methods

### 2.1. Exergy Balance

**Figure 1:**
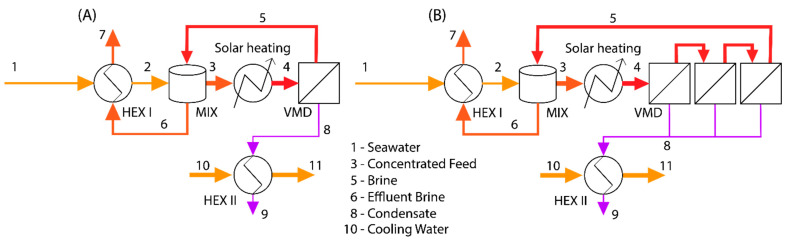
The solar thermal desalination scheme used for exergy analysis. Here, HEX I and HEX II refer to the heat exchanger on the feed and permeate stream, respectively. The MIX refers to a mixing tank used for thermal and concentration management of the solar-VMD loop. (**A**) A single-stage process. (**B**) A multi-stage process with 3 stages.

Exergy analysis is a common means of investigating the energetic performance of a process [16]. Exergy can be described as useful energy, or rather the energy available to do work [16]. Any process necessarily involves the creation of entropy or the destruction of exergy [16]. An exergy balance was developed for the solar thermal desalination process shown in Figure 1. In this process, seawater enters the system and passes through a heat exchanger where it recovers heat from the effluent brine. From the heat exchanger, it enters a mixing/buffer tank, where it is mixed with the recycled brine from the membrane module. The concentrated saltwater from this tank is then heated to the desired feed temperature via a solar collector and the feed stream enters the membrane module where it undergoes separation. The permeate vapor leaves the module and is condensed by the seawater stream in a recuperator. The brine is recycled to the mixing tank, and an effluent is drawn off from this tank to maintain a steady state within the system. A control volume was defined around the system so that the only materials crossing the boundary of the system are the cool seawater streams, the brine, and the condensed permeate. Each of these streams has an exergy term associated with them that needs to be defined. Other than the material streams, the only exergy term that crosses the boundary of the control volume is due to the energy absorbed by the solar collector. For our calculations, a basis of 1 kg h^−1^ feed flow rate was assumed along with a constant recovery ratio resulting in an effluent brine mass fraction of 0.1 (recovery ratio 0.65). This was done with the recognition that, because VMD is a modular process, the whole process can scale up or down to meet any desired capacity.

Signorato et al. defined the general exergy balance as follows [17]:

(1) $$Ex_{t}-W_{t}=\frac{d}{dt}{\left(A^{t}\right)}_{cv}+\sum_{i=1}^{\hat{N}}{\dot{m}}_{i}b_{i}^{t}+\sum_{i=1}^{\hat{N}}{\dot{m}}_{i}\xi_{0}+Ex_{irr}$$

where *Ex_t_* is the thermal exergy flux entering the system, and *W_t_* is the mechanical power added to the system [17]. *A^t^* is the total non flow exergy defined by [17]:

(2) $$A^{t}=U+p_{0}V-T_{0}S+E_{k}+E_{p}$$

where *U*, *p*_0_, *V*, *T*_0_ are, respectively, the internal energy, dead state pressure, volume, dead state temperature, and entropy of the control volume. The dead state is typically defined as the environmental conditions and is the reference state for all calculations [16]. If a system is in equilibrium with the surroundings, it is said to be at a dead state and no work of any kind can be done by the process [16]. *E_k_* and *E_p_* are the kinetic and potential energy of the control volume and *ṁ_i_* is the mass flow rate of stream “*i*” leaving the control volume. The specific flow exergy of a stream “*i*” was defined as [17]:

(3) $$b_{i}^{t}=\left(h_{i}-h_{0}\right)-T_{0}\left(s_{i}-s_{0}\right)+T_{0}{\left(\underset{k}{\sum }\hat{R_{k}}\ln\left(\frac{x_{k}}{x_{k}^{0}}\right)\right)}_{i}\;$$

where *h_i_* and *s_i_* are the specific enthalpy and entropy of stream “*i*” and *h*_0_ and *s*_0_ are the specific enthalpy and entropy of the dead state. *Ȓ_k_*, *x_k_*, and *x_k_*^0^ are the specific gas constant (J kg^−1^ K^−1^), mole fraction, and dead state mole fraction of component “*k*” in stream “*i*”. $\hat{N}$ is the number of streams entering or leaving the system. *ξ_0_* is the specific Gibb’s free energy of the dead state where *ξ_0_* = *h*_0_ − *T*s_0_ and *Ex_irr_* is the total exergy destroyed in the process.

To highlight the role of membrane parameters on the system, the exergy balance in Equation (1), with the exclusion of mechanical work, was used for the two schemes presented in Figure 1. The process is considered to be at a steady state so the transient term was neglected and the second summation term on the right-hand side of Equation (1) becomes zero (*ξ*_0_ is a constant and the sum of the mass flow rates is zero at steady state). The dead state is defined as the conditions at which stream 1 (and 10) enters the system; thus *ṁ*_1_*b*_1_*^t^* = *ṁ*_10_*b*_10_*^t^* = 0 as well. Equation (1) then becomes:

(4) $$Ex_{irr}=Ex_{solar}-{\dot{m}}_{7}b_{7}^{t}-{\dot{m}}_{9}b_{9}^{t}-{\dot{m}}_{11}b_{11}^{t}$$

where *Ex_t_* in Equation (1) is replaced by *Ex_solar_* which denotes the exergy added to the process by the solar collector defined by [10,17]:

(5) $$Ex_{solar}=Q_{solar}\left(1-\left(\frac{4}{3}\right)\left(\frac{T_{0}}{T_{sun}}\right)+\left(\frac{1}{3}\right){\left(\frac{T_{0}}{T_{sun}}\right)}^{4}\;\right)$$

where *Q_solar_* is the solar energy required to raise the temperature of stream 3 to the desired feed temperature. *T_sun_* = 6000 K was assumed as the temperature of the Sun in Equation (5). To determine *Q_solar_*, as well as the various other parameters needed for the model, a mass and energy balance was performed assuming that stream 9 leaves the system as a saturated liquid at the defined vacuum pressure, and the temperature of stream 7 is 10 K higher than that of stream 1 (an estimation of the minimum temperature difference for the heat exchanger) [18]. Thermodynamic properties of the various streams were determined using publicly available steam tables and seawater properties [19,20,21]. With all terms defined, the exergy efficiency of the process can be defined as [16,17]:

(6) $$\eta_{II}=\frac{{\dot{m}}_{7}b_{7}^{t}+{\dot{m}}_{9}b_{9}^{t}+{\dot{m}}_{11}b_{11}^{t}}{Ex_{solar}}$$

### 2.2. Geometry Definition

**Figure 2:**
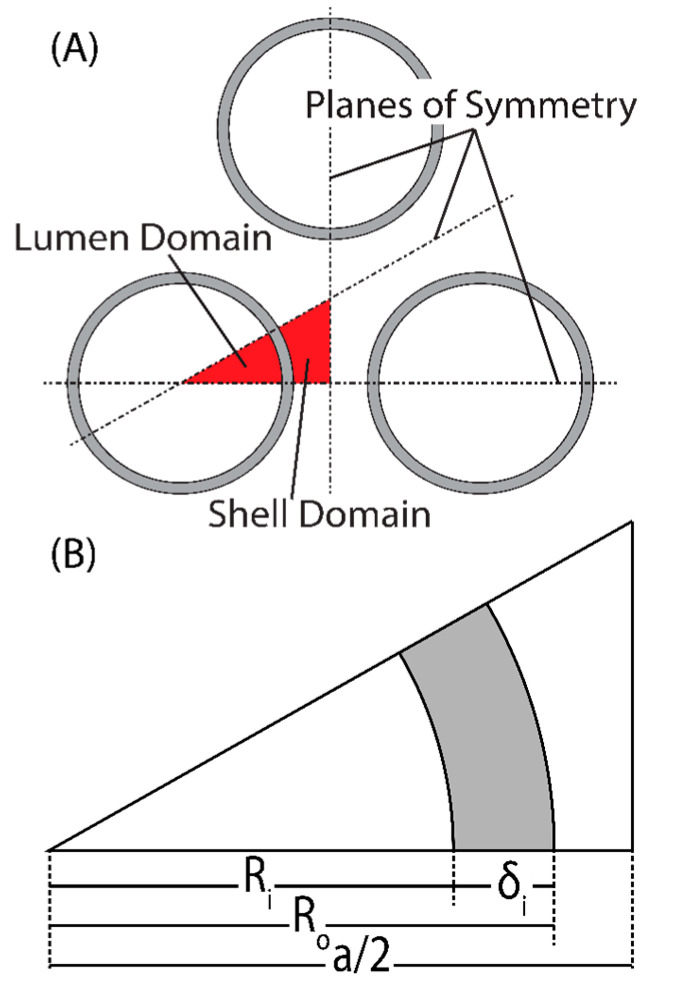
(**A**) Packing configuration showing the planes of symmetry generated (dashed lines), and the resulting lumen and shell domains used for computation. (**B**) Definition of variables for the computational domain in this work. Here, the gray-shaded areas represent porous membrane.

To describe the specific physics of the membrane module, an HFM module was modeled in COMSOL Multiphysics^®^. Figure 2 outlines the geometric definitions used to create the model. The thickness of the membrane is *δ_m_*, *R_i_* is the inner radius of the hollow fiber (the radius of the lumen), *R_o_* is the outer radius of the hollow fiber, and *a* is the fiber spacing parameter defined by *R_o_*/*a* = 0.35 (this ratio can assume any number smaller than 0.5). This packing configuration is known as “close-packed” and allows the maximum number of fibers to be fit into a module with regular spacing. The close-packed configuration generates three planes of symmetry for each fiber, which can be used to reduce the overall computational domain to a single unit cell that is descriptive of the whole. Each hollow fiber membrane consists of two domains, the saline feed, and the permeate. The permeate domain is divided into two subdomains: the membrane and the vacuum. The model was further simplified with a few assumptions: (a) momentum transfer within the vacuum domain is negligible [22]; (b) heat transfer in the permeate domain was neglected—the vacuum domain is well insulated, and conductive heat transfer through the membrane is negligible [23,24]; and (c) mass transfer within the permeate was neglected and the mass fraction of water vapor everywhere assumed to be unity—for the latter to be valid, the feed stream is assumed to be degassed. With these assumptions, the permeate flux is determined using the total pressure drop across the membrane and Darcy’s law can be used to model the flow of permeate through the membrane [25]. Because the outlet pressure at the vacuum/membrane interface is constant, the driving force in Darcy’s law is only a function of saturation pressure, which only exists at the feed/membrane interface. The computational domain can then be limited to only the feed side of the membrane, and Darcy’s law applied as a boundary condition along the membrane/feed interface. It should be noted that for a shell side feed, axial symmetry cannot be assumed and the model must be three-dimensional. For the lumen side feed, axial symmetry holds; however, a three-dimensional model was used to keep definitions consistent between the domains.

### 2.3. Momentum Transfer Governing Equations and Boundary Conditions

Momentum transfer of the feed was defined by the Navier–Stokes equations and the continuity equation [26]:

(7) $$\rho \left({\vec{u}}_{f}\cdot \nabla {\vec{u}}_{f}\right)=\nabla \cdot \left(-P_{f}\cdot \vec{\vec{I}}+\mu_{f}\left(\nabla {\vec{u}}_{f}+{\left(\nabla {\vec{u}}_{f}\right)}^{T}\right)\right)$$

(8) $$\rho \nabla \cdot {\vec{u}}_{f}$$

where *ρ* is the density of the fluid, ${\vec{u}}_{f}$ is the velocity vector, *P_f_* is the pressure of the feed fluid, *µ_f_* is the dynamic viscosity of the feed fluid, and $\vec{\vec{I}}$ is the identity tensor. At the fiber inlet, an average velocity was provided as the boundary condition: ${\vec{u}}_{f}\left(z=0\right)={\vec{u}}_{f,in}$. A no-slip boundary condition was provided at the membrane/feed interface: ${\vec{u}}_{f}\left(r=R_{i},R_{o}\right)=\vec{0}\;\left({m\;s}^{-1}\right)$. An outlet pressure was defined at the outlet of the feed channel: *P* (*z* = *L_m_*) = 1 atm.

### 2.4. Mass Transfer Governing Equations and Boundary Conditions

Based on the above assumptions, permeate flux may be defined based on Darcy’s law [25]:

(9) $$q=\frac{\kappa }{\mu }\Delta P$$

where *q* is the volumetric flow rate of the fluid, *κ* is the permeability of the membrane, *µ* is the dynamic viscosity of the fluid, and Δ*P* is the pressure drop across the membrane. The permeability of a specific membrane is difficult to find in the absence of experimental data. On this account, it is desirable to find another definition for flowrate that can be more readily adopted for modeling purposes. Zhang et al. (2015) defined the following equation for mass flux across a membrane (Equation (2)) [27]:

(10) $$N=C_{t}M_{w}\Delta P$$

where *N* is the mass flux across the membrane (kg m^−2^ s^−1^, LMH), and *M_w_* is the molar mass of water. The lumped diffusion coefficient (*C_t_*) is the sum of the Knudsen diffusion coefficient (*C*_1_) and the Poiseuille diffusion coefficient (*C*_2_). Each of these coefficients is determined using the membrane parameters (Equations (1)–(13)) [27].

(11) $$C_{t}=C_{1}+C_{2}$$

(12) $$C_{1}=\frac{4d_{p}}{3\delta_{m}\tau }{\left(\frac{1}{2\pi RM_{w}T_{f}}\right)}^{1/2}$$

(13) $$C_{2}=\frac{d_{p}\epsilon P_{m}}{32\delta_{m}\tau \mu RT_{f}}$$

where *d_p_* is the pore diameter, *δ_m_* is the thickness of the membrane, *τ* is the membrane tortuosity, *R* is the ideal gas constant, *T_f_* is the temperature, and *ϵ* is the membrane porosity (void fraction). *P_m_* is the mean pressure within the membrane defined by [27]:

(14) $$P_{m}=\frac{P_{vac}+P_{sat}}{2}$$

*P_vac_* is the vacuum pressure (5 kPa) which is in the normal range for high-production VMD [28]. *P_sat_* is the saturation pressure of water at temperature *T_f_* defined by [27]:

(15) $$P_{sat}=a_{w}\exp\left(23.238-\frac{3841}{T_{f}-45}\right)$$

The activity coefficient of water, *a_w_*, is defined based on the mole fraction of salt (*x_s_*) [29]:

(16) $$a_{w}=1-0.5x_{s}-10x_{s}^{2}$$

*a_w_* is a polynomial fitting that is accurate up to the saturation concentration of salt in water (about 350 g/L), which is valid from 30 °C to 100 °C [30]. At this concentration, *a_w_* takes on a constant value as salt spontaneously precipitates out of the solution. Finally, Δ*P* is defined simply as the difference between the saturation pressure and the vacuum pressure [27].

(17) $$\Delta P=P_{sat}-P_{vac}$$

The model from Equations (10)–(17) thus provides the basis of all calculations relating to permeate flux. A model for the binary mass transport within the feed stream was defined based on a mass balance [31]:

(18) $$\nabla \cdot {\vec{j}}_{i}+\rho \left({\vec{u}}_{f}\cdot \nabla \right)\omega_{i}=0$$

where ${\vec{j}}_{i}$ is the diffusive flux of component “*i*” and *ω_i_* is the mass fraction of component “*i*” in the fluid [31].

(19) $${\vec{N}}_{i}={\vec{j}}_{i}+\rho {\vec{u}}_{f}\omega_{i}$$

Is the total flux of component “*i*” [31].

(20) $${\vec{j}}_{i}=-\left(\rho D_{i}^{m}\nabla \omega_{i}+\rho \omega_{i}D_{i}^{m}\frac{\nabla M_{n}}{M_{n}}-{\vec{j}}_{ci}\right)$$

where *D_i_^m^* is the mixture averaged diffusion coefficient defined by [31]:

(21) $$D_{i}^{m}=\frac{\left(1-\omega_{i}\right)D_{ik}}{x_{k}}$$

In which *D_ik_* is the binary diffusion coefficient for species “*i*” in species “*k*” and *x_k_* is the mole fraction of species “*k*”. *M_n_* is the mean molar mass of the mixture defined by [31]:

(22) $$M_{n}={\left(\frac{\omega_{i}}{M_{i}}+\frac{\omega_{k}}{M_{k}}\right)}^{-1\;}$$

with *M_i_* being the molar mass of species “*i*”. ${\vec{j}}_{ci}$ is the mixture diffusion correction term defined by [31]:

(23) $${\vec{j}}_{ci}=\rho \omega_{i}\left(\frac{M_{i}}{M_{n}}D_{k}^{m}\nabla x_{k}\right)$$

The system is a binary mixture of water and salt (subscripts “*w*” and “*s*” respectively). The mass fraction of salt was defined at the inlet of the fiber, *ω_s_* (*z* = 0) = *ω_s,in_*. The mass flux of water across the membrane/feed interface was defined by the model for mass flux presented in Equations (10)–(17): ${\vec{N}}_{w}\left(\;r=R_{i},R_{o}\right)=N\left(\vec{n}\right)$ [27], where $\vec{n}$ is the normal vector pointing away from the feed stream. The inlet mass fraction of salt was defined for seawater ω_s,in_ = 0.035, and the diffusion coefficient for salt in water was estimated as D_sw_ = 10^−10^ m^2^ s^−1^ [32,33]. Along the boundaries not described as having boundary conditions, planes of symmetry were defined in accordance with Figure 2.

### 2.5. Heat-Transfer-Governing Equations and Boundary Conditions

The governing equations for heat transfer were defined by the heat balance [26]:

(24) $$\rho C_{p}{\vec{u}}_{f}\cdot \nabla T_{f}+\nabla \cdot {\vec{q}}_{f}=0$$

(25) $${\vec{q}}_{f}=-k_{f}\nabla T_{f}$$

where *C_p_* is the heat capacity of the fluid, ${\vec{q}}_{f}$ is the conductive heat flux, and *k_f_* is the thermal conductivity of the feed. A feed temperature served as the boundary condition at the inlet to the fiber: *T_f_* (*z* = 0) = *T_f,in_*. Heat flux across the membrane/feed interface was defined based on the mass flux in Equation (10) [34]: *q_m_* (*r* = *R_i_,R_o_*) = −*N·*H_vap_ − *h_m_* (*T_f,in_* − *T_m_*), where *q_m_* is the heat flux across the boundary, *H_vap_* is the heat of vaporization of water (40 kJ mol^−1^), *h_m_* is the convective heat transfer coefficient, and *T_m_* is the temperature at the boundary. The convective term in the heat flux boundary condition was seen to be essentially zero and was neglected [23,24]. Table 1 shows all of the input parameters used for the geometric definitions and physics calculations.

## 3. Results

### 3.1. Effects of Different Membrane Parameters

#### 3.1.1. Effect of Fiber Length

**Figure 3:**
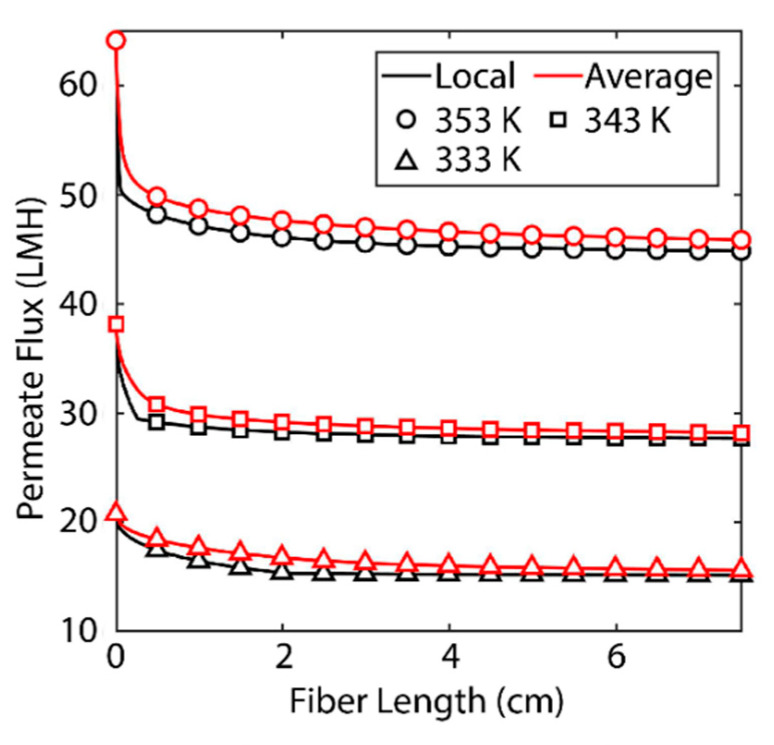
Localized permeate flux and average flux as a function of fiber length. Black lines indicate localized values for permeate flux at a certain distance from the fiber inlet. Red lines indicate the average permeate flux for a fiber with the length shown. The result is based on a shell-side feed, inner radius 350 µm, thickness 300 µm, *R*/*a* 0.35, feed velocity 5 m/s, vacuum pressure 5 kPa, tortuosity 2, pore diameter 400 nm, and porosity 0.5.

Figure 3 shows the effect of fiber length on permeate flux. The black lines indicate the localized value for permeate flux at a certain distance from the fiber inlet. The red lines indicate the average flux along an entire fiber of that length. One should observe the decrease in both local and average values of the permeate flux as fiber length increases. For a feed temperature of 353 K at the given membrane conditions the permeate flux at the inlet to the fiber can be expected to be around 65 LMH but at the outlet of a 7.5 cm fiber, the permeate flux is reduced to around 45 LMH. The cause of this degradation is polarization of temperature and concentration at the membrane/feed interface [13]. As water and heat transfer through the membrane, a boundary layer is formed in the feed stream where salt becomes more concentrated and the temperature is reduced [13]. According to Equations (15) and (16), a reduction in temperature and an increase in salt concentration leads to a decrease in saturation pressure. A decrease in saturation pressure at the membrane boundary reduces the driving force for mass transfer in Equation (10).

These polarization phenomena are important for a number of reasons. As Figure 3 shows, increasing the feed temperature also increases the magnitude of this drop in flux. This is for the same reason as noted above: reduction in temperature, by Equation (15), leads to a reduction in saturation pressure and thereby permeate flux. The boundary conditions considered along the membrane interface for heat and mass transfer are defined by the magnitude of the permeate flux. The smaller the permeate flux (${\vec{N}}_{w}$), the less heat is transferred (*q_m_*). The phenomena that lead to the boundary layer are less significant at lower temperatures, so the boundary layer is less pronounced. This becomes significant when we consider the energetic requirements of membrane distillation. MD relies on thermal energy provided to the system to evaporate water [13]. The energy requirement is significant due to both the high heat capacity of water (which makes raising the temperature difficult) and the high heat of vaporization [7]. This severely limits the efficiency of the process and makes fiber length a key parameter for design and optimization. These findings suggest that shorter membranes, which may be able to operate without a fully formed boundary layer, are desirable.

#### 3.1.2. Effect of Porosity

**Figure 4:**
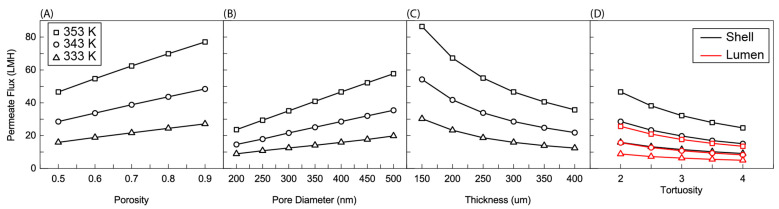
Average permeate flux as a function of (**A**) porosity, (**B**) nominal pore size, (**C**) membrane thicknesses, and (**D**) effective tortuosities. In (**A**–**C**) the feed was charged to shell side, (**D**) Shows for both cases, charging feed into the shell and lumen. Here, the *R*/*a* ratio is fixed at 0.35, the membrane length is 5 cm, feed velocity is 5 m/s, and the permeate pressure is 5 kPa.

Figure 4A shows the effect of membrane porosity on the average permeate flux of a 5 cm membrane. Porosity is defined by the void fraction of a membrane (or the volume of a membrane that is not occupied by the membrane material). Equations (11)–(13) predict a linear relationship between porosity and permeate flux. This predicted relationship is largely maintained in the average flux. Permeate flux increases approximately linearly with porosity. The slope of this linear relationship is determined by feed temperature. Lower feed temperatures show a smaller slope than higher feed temperatures. This is because Δ*P* and *P_m_* in Equations (10) and (13) are larger. The same change in porosity leads to a larger change in the overall slope determined by Equations (10)–(13). A slight concavity is present in the data sets due to polarization. Higher values of permeate flux at the inlet lead to more significant polarization and a degradation in average flux, though the initial behavior predicted by Equations (10)–(13) can be maintained.

#### 3.1.3. Effect of Pore Diameter

Pore diameter, like porosity, maintains a linear relationship with average flux due to its presence in the numerator of Equations (12) and (13) (Figure 4B). Like porosity, a larger pore diameter provides more space for vapor to transport through the membrane and therefore increases flux. Unlike porosity, pore diameter does not have an upper limit, as long as the liquid entry pressure is not reached [35]. Each of these parameters achieved around a 35 LMH increase in flux at 353 K feed temperature over the range of parameters tested.

#### 3.1.4. Effect of Thickness

The effect of thickness is again predicted well by Equations (12) and (13) (Figure 4C). The permeate flux largely follows an inverse proportionality with membrane thickness. Membrane thickness is integral to the determination of driving force. The pressure difference across the membrane is the driving force in Equation (10). As the length across which the driving force is measured decreases, flux increases. The limit to membrane thickness is the mechanical strength of the membrane. Thinner membranes are weaker and less able to withstand the application of a vacuum.

#### 3.1.5. Effect of Tortuosity

Figure 4D shows the effect of tortuosity on membrane performance. Tortuosity occurs in the denominator of both Equations (12) and (13), and this inverse exponential relationship is reflected in Figure 4D. Permeate flux decreases with an increase in tortuosity. Tortuosity is a measure of how far from a linear path a water molecule must stray in order to travel through the membrane due to membrane structure. If, on average, a water molecule can travel through the membrane while never deviating from the shortest route, the tortuosity is 1. Higher tortuosities are indicative of more “winding” paths. As above, the effect of this change in tortuosity is more dramatic for higher feed temperatures due to the larger Δ*P* and *P_m_* terms.

Figure 4D also shows a comparison between a lumen and a shell-side feed. The lumen-side feed sees significantly lower average permeate flux compared to a shell-side feed. This is because of the buildup of salt along the membrane surface. In a cylindrical geometry, mass transfer occurs more quickly in the direction of increasing radius than it does the direction of decreasing radius. This is because as the radius increases, the surface area for mass transfer steadily increases, while as radius decreases, surface area decreases. As such, when salt concentration builds up on the membrane surface, it can more readily diffuse away from the membrane in a shell-side feed than a lumen-side feed. Equations (10)–(13) also show that the driving force should be the same for each feed configuration. Because the average for permeate flux was taken at the outer surface of the membrane (shell side surface), the same driving force was divided over a relatively larger area for the lumen, which also led to the decrease shown in Figure 4D.

### 3.2. Limiting Phenomena

**Figure 5:**
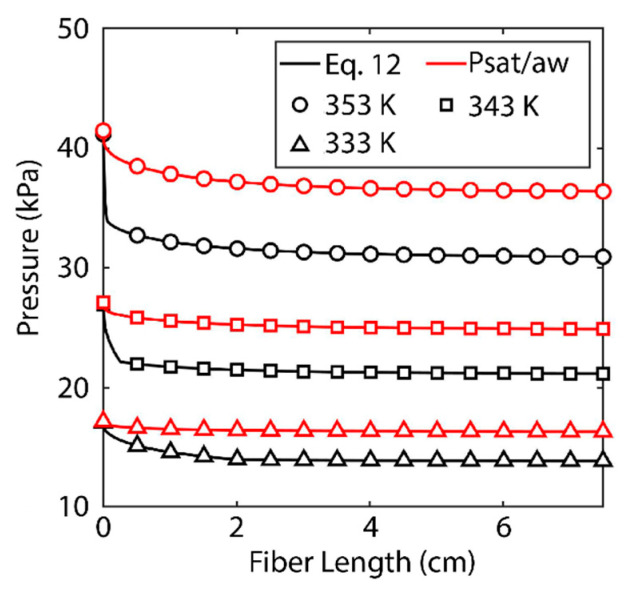
Saturation pressure as a function of fiber length. Black lines indicate saturation pressure as calculated using Equation (16). Red lines indicate saturation pressure as determined using Antoine’s equation This study is based on shell-side feed. Here, the inner radius of the hollow fiber is 350 µm and the thickness of media is 300 µm. The *R*/*a* was kept at 0.35, the fibers’ lengths are 5 cm, the feed is velocity 5 m/s, and the pressure in permeate channel was kept at 5 kPa. We assumed membrane tortuosity of 2, pore size diameter of 400 nm, and a porosity 0.5 of for the membrane media.

As noted, polarization within the boundary layer occurs as a result of a reduction in temperature and an increase in salt concentration [13]. A comparison must be made between the two phenomena to find the determining factor. Figure 5 shows the saturation pressure of water at the surface of the membrane under two conditions. The black lines indicate the actual saturation pressure calculated by Equation (15). The red lines indicate the saturation pressure based solely on Antoine’s equation (defined as *P_sat_*/*a_w_*). The red lines are only a function of temperature, while the black lines are a function of both temperature and salt concentration. The difference in the two functions is the effect of salt concentration.

While both phenomena contribute to the reduction in saturation pressure, the increase in salt concentration along the membrane surface is most significant. This can be most readily observed at the 333 K feed temperature. The reduction of Antoine’s equation is almost negligible, while the saturation pressure has a noticeable reduction. At each of the three feed temperatures, the saturation pressure decreases most significantly towards the inlet of the fiber as salt concentration increases. At a certain point, the salt concentration within the boundary layer reaches saturation and cannot increase further. At this point only does the contribution of temperature become visible. From that point on, the saturation pressure behaves as a vertical translation of Antoine’s equation. At 353 K, this saturation is reached almost immediately. At 343 K, the saturation concentration is reached after 2 mm, and at 333 K the phenomenon occurs more gradually, and saturation within the boundary layer is reached at 2 cm. These effects can also be observed in the local permeate and average permeate flux values in Figure 3. The majority of the loss of average and local permeate flux occurs towards the inlet of the fiber until the concentration of salt reaches saturation and the degradation of flux is more gradual for the remainder of the fiber’s length.

To quantify the significance of salt polarization relative to mass, one must observe the respective diffusivities. The mass diffusivity of salt in water is known to be on the order of 10^−10^ m^2^ s^−1^ [33], while the thermal diffusivity of water is on the order of 10^−7^ m^2^ s^−1^ [36]. The three orders of magnitude difference between these numbers is reflected in the results shown in Figure 5, confirming salt concentration must be the limiting factor. Salt concentration is significant in another way. Scaling is known to be a problem in desalination systems [13]. As concentration in the boundary layer increases, so does scaling [11,23,24,28]. As crystalline salt forms on the surface of the membrane, it can block pores and inhibit permeate flux. Scaling also increases the wettability of a membrane, which means the process may need to be run at a higher pressure in the vacuum domain or risk contaminating the permeate [37]. The model presented here is limited in that it does not present a kinetic model for scaling and cannot take into account how scaling will change membrane performance. Unlike salt, temperature does not have a saturation value that limits its effect on membrane performance. As length increases, the temperature boundary layer becomes more apparent and will eventually become the determining factor in membrane performance.

This effect of salt concentration polarization is important also when we consider another application of VMD. RO desalination plants are limited in their recovery because RO cannot operate above certain salinities (around a mass fraction of about 0.08) [7]. RO reaches this limit because the supply pressure necessary to overcome the osmotic pressure at these salinities is beyond the pressure that the system can physically handle or the salinity is too high for the membrane to separate [38,39]. Environmental concerns make zero-liquid-discharge systems (systems that recover all of the available water from a system with no effluent brine) highly attractive [7,40]. VMD is a phenomenal candidate for such systems because, as Figure 4 and Figure 5 show, the membrane is still able to achieve very high flux despite operating at the saturation point of salt in water (at least at the membrane/feed interface). VMD is then able to remove all water from the saline feed and crystalize the remaining salt under zero liquid discharge conditions [41].

### 3.3. Effect of Baffling Design

**Figure 6:**
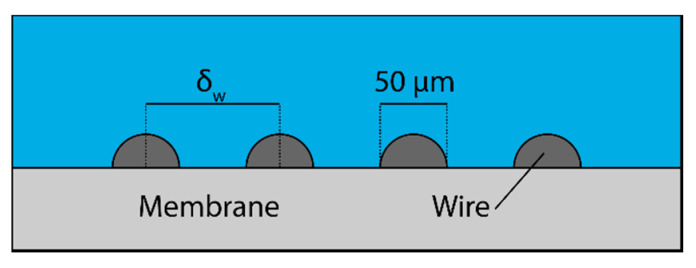
Baffling geometry that was used to reduce concentration polarization on the surface of membranes.

One possible means of ameliorating the problem of polarization is to add some sort of baffling to the surface of the membrane [13,14]. Figure 6 shows one such method of baffling. A thin wire can be wrapped around the membrane to induce turbulence and break the boundary layer and eliminate polarization. The spacing between coils of the wire can be controlled to improve performance.

**Figure 7:**
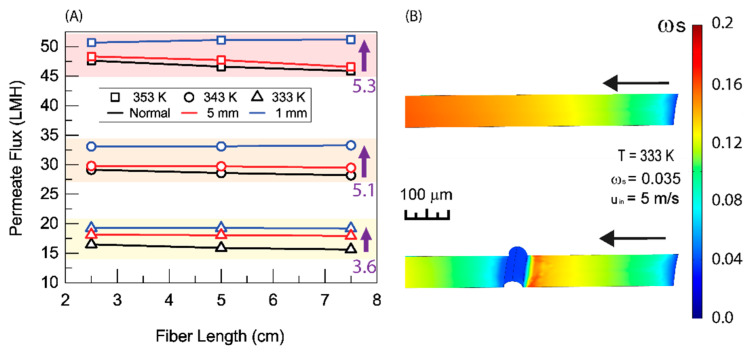
(**A**) Average permeate flux as a function of fiber length with different baffling spacings. (**B**) Salt concentration profile along he membrane surface for a normal (unbaffled) fiber and a fiber with baffling at 1 mm spacing. The result is based on the shell side feed, inner radius 350 µm, thickness 300 µm, *R*/*a* 0.35, feed velocity 5 m/s, vacuum pressure 5 kPa, tortuosity 2, pore diameter 400 nm, and porosity 0.5.

The addition of this simple baffling scheme leads to a noticeable improvement in membrane permeate flux. Figure 7 shows a comparison of baffled and unbaffled fibers. For the 5 mm spacing, the improvement is most significant at a 333 K feed temperature. As temperature increases the improvement is reduced. This can be explained by Figure 6. At each coil, the boundary layer is broken and the conditions at the surface of the membrane return to approximately what they were at the inlet (or that of the bulk fluid). At both the 343 K and 353 K feed temperatures, the concentration of salt reaches saturation before the first coil. At these feed temperatures, salt reaches saturation within the boundary layer too quickly for the 5 mm spacing to be effective; however, the 333 K feed temperature solutions never reach saturation under the baffling scheme devised. For the 1 mm coil spacing at each feed temperature, a significant increase in permeate flux was observed. In Figure 3 and Figure 4, it can be observed that, while the membrane parameters allow for a very high flux at the inlet, for the average flux to achieve a similarly high value, the membrane needs to be short. By adding this baffling scheme, the concentration profile, and therefore the permeate flux profile, of a much shorter membrane is repeated along the entire length of the membrane. Shortening the distance between the baffles shortens the length of membrane that is repeated and increases flux even further.

There is a twofold problem with this scheme. First, the addition of the wire around the membrane covers a portion of the membrane and reduces the surface area of the membrane available for distillation. This is exemplified by the 1 mm spaced baffling in Figure 7. At the highest temperatures, the shortened baffling leads to the most significant increase in permeate flux. At lower temperatures, the improvement is less pronounced. Decreasing the spacing at these temperatures covers more membrane area and reduces the actual surface area for mass transport. Secondly, the sharp, local, increase in salt concentration on the upstream side of the wire will result in an increase in scaling at that point. The first problem can be readily optimized to achieve better results. The second problem can be handled by the addition of hydrophobic or omniphobic coatings to the surface of the membrane and whatever baffling is added to it [7]. Reducing the distance between baffles to reduce salt concentration will also help to inhibit scaling by reducing the concentration of salt along the boundary.

### 3.4. Exergy Efficiency

The effect of membrane parameters on exergy efficiency of the solar-thermal desalination system described in Figure 1 was computed using a mass and energy balance and Equations (1)–(5). Table 2 shows the mass and energy balance of one of these solutions. The recovery ratio calculated by the mass flux values shown in Figure 4 is low (single-pass recovery ratio on the order of 10^−4^). In comparison to this single pass recovery ratio, the overall recovery ratio of the system shown in Figure 1 is designed to be 0.65. To make up the difference between the single-pass recovery ratio and the overall recovery ratio, the streams that comprise the recycling (streams 3, 4, and 5) must be substantially larger than the streams outside of it. The magnitude of these streams means they dominate the mass and energy balance calculations. Another effect of the desired recovery ratio is that the feed stream (stream 1) cannot provide sufficient cooling duty to condense the permeate stream (stream 8). To condense the permeate, another stream (stream 10) with a higher flow rate must provide the cool water to condense the permeate. Finally, the magnitude of the recycle streams relative to other streams means that the properties of the recycle loop are roughly the same and are largely unaffected by the feed being added or the effluent or permeate removed.

**Figure 8:**
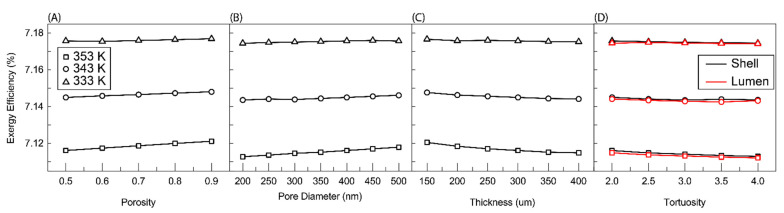
Exergy efficiency as a function of (**A**) porosity, (**B**) nominal pore size, (**C**) membrane thicknesses, and (**D**) effective tortuosities. In (**A**–**C**) the feed was charged to shell side, (**D**) Shows for both cases, charging feed into the shell and lumen. Here, the *R*/*a* ratio is fixed at 0.35, the membrane length is 5 cm, feed velocity 5 m/s, and the channel in the permeate pressure is maintained at 5 kPa.

Figure 8 shows the effect of membrane parameters on exergy efficiency. Three things are immediately observable from these plots. The first is that increasing temperature decreases exergy efficiency. This is likely due to the increased heating duty required of the solar collector and the resultant increase in *Q_solar_* and *Ex_solar_*. The effect of temperature on the other three streams that determine exergy efficiency in Equation (6) is less significant. No change should occur in stream 7 because the recovery ratio is fixed and the outlet temperature of stream 7 is determined by the feed temperature of stream 1. Stream 11 should see an increase in temperature due to the increased temperature of stream 8. This should result in an increase in exergy efficiency but the flow rate of streams 3 and 4, which determine the heat duty, are orders of magnitude higher than these streams and therefore the effect of temperature is most pronounced on the increased heating duty required. The second phenomenon to be observed is how little effect membrane parameters has on exergy efficiency. The largest change occurs for thickness at 353 K. Around a 0.005% decrease is observed by increasing the thickness of the membrane. This again is likely due to the relative magnitude of *Q_solar_* to the membrane parameters and its dominating effect on Equation (6). The final phenomenon is the functionality of exergy efficiency with the respective membrane parameters. The trends are largely the same as those observed for permeate flux in Figure 4 albeit of a very depressed nature. This is probably the remaining effect of streams 8–11 observed above. Increasing flux increases the flow rate of streams 8 and 9 and with it the exergy leaving with stream 9. The increase in flow rate also leads to an increase in the temperature of stream 11 and its exergy.

**Figure 9:**
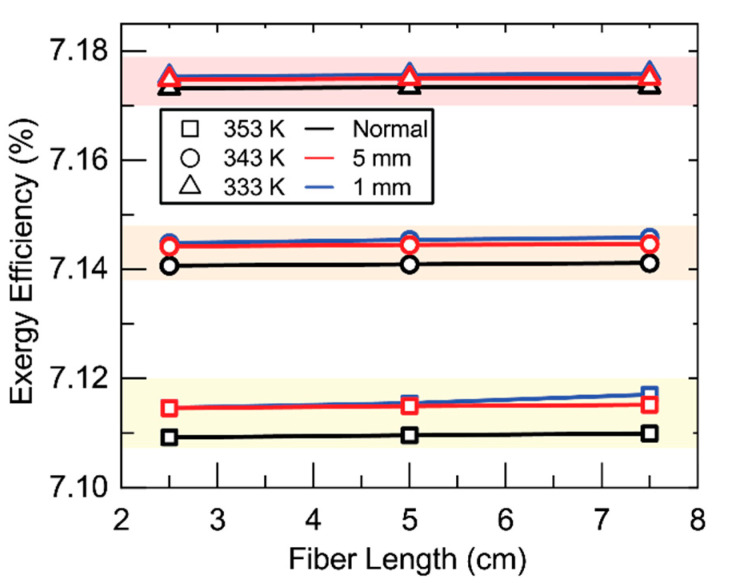
Exergy efficiency as fiber length changes at various baffling spacings. Shell-side feed. Inner radius 350 µm, thickness 300 µm, *R*/*a* 0.35, feed velocity 5 m/s, vacuum pressure 5 kPa, tortuosity 2, pore diameter 400 nm, porosity 0.5.

Figure 9 shows the effect of baffling on exergy efficiency. The effect of baffling is, like membrane parameters, essentially negligible on exergy efficiency. A small effect can be observed wherein the addition of baffling increases exergy efficiency. This follows the same reasoning as membrane parameters and permeate flux in Figure 8. The addition of baffling also increases temperature along the interface by mixing with the bulk. The higher temperature along the interface results in an increased temperature of stream 8 and therefore temperature and exergy of stream 11.

**Figure 10:**
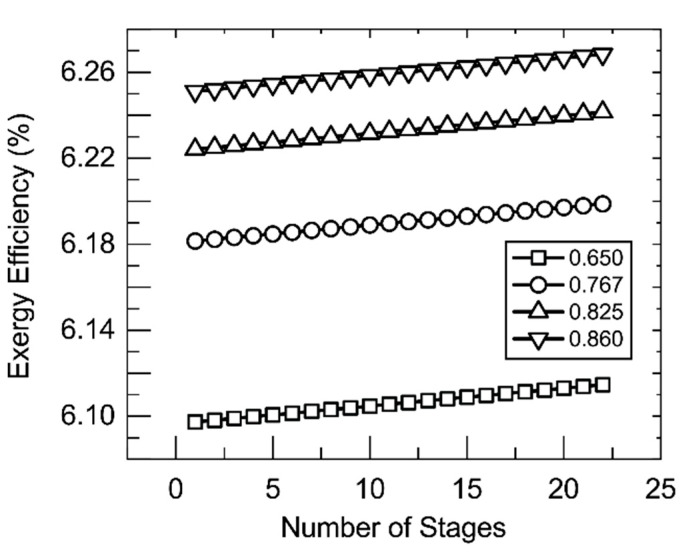
Exergy efficiency as the number of stages increases. Shell-side feed. Inner radius 350 um, thickness 300 um, *R*/*a* 0.35, feed velocity 5 m/s, vacuum pressure 5 kPa, tortuosity 2, pore diameter 400 nm, porosity 0.5, and a value for the ṁ_11_ = 200 kg/hr. Legend indicates recovery ratio. Recovery ratio 0.65 corresponds to mass fraction of 0.1 for the salt in stream 7.

The insight provided by these findings is that they suggest that membrane parameters are largely insignificant to the exergetic performance of the system. It is important to note that this design is for a single module with a limited single-pass recovery ratio. A multiple-effect system (as shown in Figure 1B) in place of the single membrane module should be able to achieve a much higher single-pass recovery ratio that may have a greater effect on exergy efficiency. Figure 10 shows the effect of such a multiple-effect scheme. In this scheme, membrane modules like that examined in Figure 4 and Figure 8 are placed in series. Increasing the number of modules in the series leads to an increase in recovery ratio and, with it, exergy efficiency. Normally, multiple effect configurations also contain an internal mode of heat recovery from stage to stage; however, such a recovery system was not considered in these calculations and may be effective in increasing exergy efficiency.

Figure 10 also shows the effect of overall recovery ratio on exergy efficiency. Increasing recovery ratio increases exergy efficiency due to an increase in the flow rate of stream 9 and the temperature and exergy of stream 11. This increase is still predominantly marginal from an overall exergy efficiency standpoint; however, it is significant to note the effect that the exergy of stream 9 can have on the efficiency of the process. A great amount of exergy leaves the system as evinced by the change observed in exergy efficiency with the stream’s increase. Recovery and reuse of this exergy can be valuable for increasing the economic viability of solar thermal membrane distillation processes, particularly of high-recovery systems such as the one described here. A problem for optimization remains here. The addition of more stages, while increasing exergy efficiency as shown, leads to increased cost. It may be that the addition of these stages, from a cost standpoint, does not affect a significant enough increase in exergy efficiency to be practical.

## 4. Conclusions

The results suggest that for the high permeance of water vapor in a VMD module, concentration polarization within the boundary layer becomes the limiting phenomenon. The increase in salt concentration at the membrane interface significantly lowers the vapor pressure of feed water, which provides the driving force for permeate flux. The reduction of permeate flux could be mitigated by the application of a baffling scheme to the surface of the membrane. This baffling scheme improves permeate flux by breaking the boundary layer and inducing turbulence into the feed stream, thus allowing for increased water recovery at a given input energy. However, the increase in the recovery ratio led only to a small increase in exergy efficiency. This increase was more pronounced when multiple stages of VMD modules were utilized. From an exergetic standpoint, membrane characteristics and module design are of little concern to the design of a vacuum membrane distillation process. Far more important is improvement in solar energy collection and process designs that enable that energy to be effectively reused. Within the membrane module itself, scaling is the most important factor. Our model showed that highly permeable membranes can be expected to operate near saturation for almost their entire length. Hence, reducing a membrane’s propensity for scaling becomes important from both operational and cost standpoints.

## Figures and Tables

**Table 1:** Operating parameters and constants.

Parameter	Value	Parameter	Value
$T_{f,in}$	333–353 K	$d_{p}$	200–500 nm
$P_{vac}$	5 kPa	τ	2–4
$u_{f,in}$	5 m s^−1^	ϵ	0.5–0.9
$R_{i}$	350 μm	$L_{m}$	2.5–7.5 cm
$\delta_{m}$	150–400 μm	$\omega_{s,in}$	0.35

**Table 2:** Mass and energy balance, shell side feed. Inner radius 350 um, thickness 300 um, *R*/*a* 0.35, Length 5 cm, feed velocity 5 m/s, vacuum pressure 5 kPa, tortuosity 2, pore diameter 400 nm.

Scheme	Mass Flow Rate (kg h^−1^)	Mass Fraction of Salt %	Temperature (K)
1	1	3.5	288
2	1	3.5	307.2
3	2041.3	10	352.8
4	2041.3	10	353
5	2040.7	10.003	352.8
6	0.35	10	352.8
7	0.35	10	298
8	0.65	0	350.5
9	0.65	0	306
10	50	3.5	288
11	50	3.5	295.8

## Data Availability

All codes and files used to generate the presented figures and data will be made available to the journal upon request.

## Nomenclature

Terms	Definition
$\delta_{m}$	Membrane thickness (m)
ϵ	Membrane porosity
$\eta_{II}$	Exergy efficiency
κ	Membrane permeability $\left(m^{2}\right)$
μ	Dynamic viscosity of fluid (Pa s)
$\mu_{f}$	Dynamic viscosity of the feed stream (Pa s)
$\xi_{0}$	Specific Gibbs free energy of the dead state $\left({J\;\mathrm{kg}}^{-1}\right)$
ρ	Density of feed stream $\left({\mathrm{kg}\;m}^{3}\right)$
τ	Membrane tortuosity
$\omega_{i}$	Mass fraction of component “i”
$\omega_{s,in}$	Mass fraction of salt at the inlet
a	Fiber spacing parameter
$a_{w}$	Activity coefficient of water
$A^{t}$	Total non-flow exergy (J)
$b_{i}^{t}$	Specific flow exergy of stream “i” $\left({J\;\mathrm{kg}}^{-1}\right)$
$C_{1}$	Knudsen diffusion coefficient $\left({s\;\mathrm{mol}\;\mathrm{kg}}^{-1}m^{-1}\right)$
$C_{2}$	Poiseuille diffusion coefficient $\left({s\;\mathrm{mol}\;\mathrm{kg}}^{-1}m^{-1}\right)$
$C_{p}$	Heat capacity of fluid $\left({J\;\mathrm{kg}}^{-1}K^{-1}\right)$
$C_{t}$	Total diffusion coefficient $\left({s\;\mathrm{mol}\;\mathrm{kg}}^{-1}m^{-1}\right)$
$d_{p}$	Mean pore diameter (m)
$D_{i}^{m}$	Mixture average diffusion coefficient of component “*i*” $\left(m^{2}{\;s}^{-1}\right)$
$D_{sw}$	Diffusion coefficient for salt in water $\left(m^{2}{\;s}^{-1}\right)$
$E_{k}$	Kinetic energy (J)
$E_{p}$	Potential energy (J)
$Ex_{irr}$	Total exergy loss due to system irreversibility (W)
$Ex_{solar}$	Solar exergy flux (W)
$Ex_{t}$	Total Thermal Exergy Flux (W)
$h_{0}$	Specific enthalpy of dead state $\left({J\;\mathrm{kg}}^{-1}\right)$
$h_{i}$	Specific enthalpy of stream “*i*” $\left({J\mathrm{kg}}^{-1}\right)$
$\hat{h_{m}}$	Convective heat transfer coefficient $\left({\mathrm{Wm}}^{-2}K^{-1}\right)$
$H_{vap}$	Heat of vaporization of water $\left({\mathrm{kJ}\;\mathrm{mol}}^{-1}\right)$
$\vec{\vec{I}}$	Identity tensor
${\vec{j}}_{ci}$	Mixture diffusion correction term $\left({\mathrm{kg}\;m}^{-2}s^{-1}\right)$
${\vec{j}}_{i}$	Diffusive flux of component “i” in the feed stream $\left({\mathrm{kg}\;m}^{-2}s^{-1}\right)$
$k_{f}$	Thermal conductivity of feed stream $\left({W\;m}^{-1}K^{-1}\right)$
$L_{m}$	Length of membrane module (m)
${\dot{m}}_{i}$	Mass flowrate of stream “i” $\left({\mathrm{kg}\;s}^{-1}\right)$
$M_{i}$	Molar mass of component “i” in membrane feed $\left({\mathrm{kg}\;\mathrm{mol}}^{-1}\right)$
$M_{n}$	Mean molar mass of the feed stream $\left({\mathrm{kg}\;\mathrm{mol}}^{-1}\right)$
n	Number of fibers in a module
$\vec{n}$	Normal vector
N	Mass flux of water vapor across membrane $\left({\mathrm{kg}\;m}^{-2}s^{-1}\right)\;$
$\hat{N}$	Total Number of Streams entering and leaving
${\vec{N}}_{i}$	Total mass flux of component “*i*” within the membrane feed stream $\left({\mathrm{kg}\;m}^{-2}s^{-1}\right)$
${\vec{N}}_{w}$	Total mass flux of water within the membrane feed stream $\left({\mathrm{kg}\;m}^{-2}s^{-1}\right)$
$p_{0}$	Dead state pressure (Pa)
$P_{f}$	Pressure of the feed stream (Pa)
ΔP	Transmembrane change in pressure (Pa)
$P_{m}$	Average pressure within the membrane (Pa)
$P_{sat}$	Saturation pressure of water (Pa)
$P_{vac}$	Vacuum pressure (Pa)
q	Volumetric flowrate $\left(m^{3}{\;s}^{-1}\right)$
${\vec{q}}_{f}$	Conductive heat flux in the membrane feed stream $\left({W\;m}^{-2}\right)$
$q_{m}$	Transmembrane heat flux $\left({W\;m}^{-2}\right)$
$Q_{solar}$	Solar energy (W)
r	Radial spatial variable (m)
R	Ideal gas constant $\left({J\;\mathrm{mol}}^{-1}K^{-1}\right)$
$R_{i}$	Inner radius of the membrane (m)
$\hat{R_{k}}$	Specific Gas constant for component “k” $\left({J\;\mathrm{kg}}^{-1}K^{-1}\right)$
$R_{o}$	Outer radius of membrane (m)
$s_{i}$	Specific entropy of stream “*i*” $\left({J\;\mathrm{kg}}^{-1}K^{-1}\right)$
$s_{0}$	Dead state-specific entropy $\left({J\;\mathrm{kg}}^{-1}K^{-1}\right)$
S	Entropy $\left({J\;K}^{-1}\right)$
$T_{0}$	Dead State temperature (K)
$T_{f}$	Feed stream temperature (K)
$T_{f,in}$	Feed temperature at inlet (K)
$T_{m}$	Feed stream temperature at membrane interface (K)
$T_{sun}$	Temperature of the Sun (K)
${\vec{u}}_{f}$	Feed velocity $\left({m\;s}^{-1}\right)$
${\vec{u}}_{f,in}$	Feed velocity at inlet $\left({m\;s}^{-1}\right)$
U	Internal Energy (J)
V	Volume $\left(m^{3}\right)$
$W_{t}$	Mechanical Power (W)
$x_{k}$	Mole fraction of component k
$x_{k}^{0}$	Dead state mole fraction of component k
$x_{s}$	Mole fraction of salt in feed stream
z	Axial spatial variable (m)
